# Sol–Gel Synthesis and Comprehensive Study of Structural, Electrical, and Magnetic Properties of BiBaO_3_ Perovskite

**DOI:** 10.3390/gels11060450

**Published:** 2025-06-12

**Authors:** Faouzia Tayari, Kais Iben Nassar, João Pedro Carvalho, Sílvia Soreto Teixeira, Imen Hammami, Sílvia Rodrigues Gavinho, Manuel P. F. Graça, Manuel Almeida Valente

**Affiliations:** 1 I3N-Aveiro, Department of Physics, University of Aveiro, 3810-193 Aveiro, Portugal; faouzia.tayari@ua.pt (F.T.); jpfc@ua.pt (J.P.C.); silvia.soreto@ua.pt (S.S.T.); imenhammami@ua.pt (I.H.); silviagavinho@ua.pt (S.R.G.); mpfg@ua.pt (M.P.F.G.); mav@ua.pt (M.A.V.); 2 TEMA-Centre for Mechanical Technology and Automation, Department of Mechanical Engineering, University of Aveiro, 3810-193 Aveiro, Portugal

**Keywords:** sol–gel synthesis, structural characterization, electrical conductivity, magnetic properties, multifunctional materials

## Abstract

In this study, BiBaO_3_ perovskite was successfully synthesized via the sol–gel method and thoroughly characterized to evaluate its structural, microstructural, dielectric, electrical, and magnetic properties. X-ray diffraction (XRD) confirmed the formation of a single-phase perovskite structure with high crystallinity. Scanning electron microscopy (SEM) coupled with energy-dispersive X-ray spectroscopy (EDX) revealed a uniform grain morphology and elemental composition consistent with the intended stoichiometry. Dielectric measurements exhibited strong frequency-dependent behavior, suggesting potential for capacitive applications. The electrical conductivity displayed thermally activated behavior, indicative of semiconducting characteristics. Magnetic measurements showed weak ferromagnetic behavior at room temperature, an unusual observation for undoped BaBiO_3_-based systems. This magnetism may stem from subtle structural distortions or compositional variations introduced during synthesis. Comparison with previously reported studies underscores the significant influence of the synthesis route and microstructural features on the multifunctional properties of BiBaO_3_. Overall, the results highlight the promise of sol–gel-derived BiBaO_3_ as a candidate for multifunctional electronic and magnetic applications.

## 1. Introduction

In recent years, the pursuit of multifunctional materials has intensified due to growing demands from smart technologies, sustainable energy, and high-performance electronics [1,2,3]. Perovskite ceramics have emerged as leading candidates because of their unique structural flexibility, thermal stability, and tunable electrical and magnetic properties [4,5]. Advanced synthesis approaches, such as in situ chlorination and green fabrication methods, have enabled breakthroughs in optoelectronics, including efficient deep-blue LEDs and fingerprint visualization devices [6,7]. At the same time, researchers have developed novel porous structures and dual-scale aerogels with impressive electromagnetic absorption capabilities, expanding the scope of multifunctional ceramic applications [8,9]. Modeling techniques using meshless and thermodynamic approaches further enhance the understanding of nonlinear wave behavior and particle interactions within complex material systems [10]. Environmental impact and resource sustainability have become essential considerations in materials design. Studies on embodied CO_2_ in trade [6], deforestation avoidance [7], and heavy metal transport in soils [11] demonstrate the importance of integrating ecological concerns into materials development. The reuse of industrial wastes particularly red mud and fly ash in geopolymer composites supports both sustainability and material performance, showing effectiveness in pollutant remediation and heavy metal immobilization [12,13,14,15]. Further work has introduced thermodynamic models that account for particle breakage, crystallization, and ion migration under harsh conditions, improving the reliability of ceramics in real-world environments [16,17,18,19]. These materials also show strong potential in contaminated soil treatment through flowing-water remediation and unified chemical biological technologies [15]. Recent multi-field models for saline soils reinforce the role of simulation in advancing sustainable ceramic systems [20,21].

Perovskite materials with the general formula ABO_3_ have attracted widespread research interest due to their exceptional range of physical and chemical properties [22,23]. These oxides often exhibit multifunctional characteristics, including ferroelectricity, magnetoresistance, superconductivity, and high dielectric constants, which make them valuable for a wide spectrum of technological applications such as capacitors, sensors, memory devices, and spintronic components [24,25,26,27,28]. One of the significant advantages of the perovskite structure is its ability to accommodate a wide variety of cations at the A and B sites, enabling extensive chemical substitution and property tuning. Bismuth-based perovskites are of particular interest because of the unique electronic configuration of the Bi^3+^ ion, which contributes to complex electronic and magnetic interactions. Among them, BaBiO_3_ has been studied extensively and is known for its insulating nature, which arises from charge disproportionation between Bi^3+^ and Bi^5+^ ions [29]. This unusual electronic structure gives rise to intriguing phenomena, such as polaron formation, breathing-mode lattice distortions, and, under appropriate doping conditions, superconductivity [30,31,32].

Several researchers have reported superconducting behavior in Ba_1−x_K_x_BiO_3_ and BaPb_1−x_Bi_x_O_3_ systems with transition temperatures up to 30 K [33,34,35]. These materials are among the earliest examples of non-cuprite oxide superconductors. Moreover, BaBiO_3_ and its derivatives explored for photocatalytic applications, with Bi^3+^ playing a role in facilitating visible-light absorption due to 6s^2^ lone pair effects. Although BiBaO_3_ has received significant attention, reports on BiBaO_3_ in the reverse stoichiometry are non-existent, due to challenges in stabilizing the structure or limited exploration in the literature. Investigating BiBaO_3_ may reveal new structural or functional characteristics not observed in the traditional BaBiO_3_ compound [36]. The synthesis method plays a crucial role in determining the structural integrity and properties of perovskite materials. Traditional solid-state synthesis methods require elevated temperatures and often result in inhomogeneous products and large particle sizes. In contrast, the sol–gel method provides better control over stoichiometry, particle morphology, and lower crystallization temperatures. This approach enables molecular-level mixing of precursors and often leads to improved material properties [37].

An array of studies has demonstrated the advantages of the sol–gel method in preparing perovskites. For example, LaFeO_3_ synthesized using the sol–gel route and reported enhanced magnetic and dielectric properties compared to solid-state methods. Similarly, material BiFeO_3_ nanoparticles prepared via sol–gel experienced increased ferroelectricity and reduced leakage current due to improved microstructural control [38]. In terms of characterization, X-ray diffraction remains the primary technique for confirming phase formation, crystal structure, and crystallite size. For perovskites, a typical observation includes sharp diffraction peaks corresponding to a cubic, tetragonal, or orthorhombic symmetry, depending on the ionic radii and distortion factors [39,40]. For example, BaBiO_3_ commonly crystallizes in a monoclinic structure at room temperature, although reports also exist of orthorhombic phases under specific conditions. Scanning electron microscopy allows for detailed visualization of the surface morphology, particle size, and porosity. The microstructure has a significant impact on dielectric and electrical behavior, with smaller grains contributing to higher dielectric constants due to increased grain boundary polarization. Energy-dispersive X-ray spectroscopy, typically coupled with SEM, helps confirm the elemental composition and uniformity across the sample [41].

Dielectric measurements provide insight into the polarizability and insulation of materials. For perovskite oxides, high dielectric constants are often observed, especially in systems where lattice distortions or dipolar defects contribute to polarization mechanisms [42,43]. Frequency-dependent dielectric studies are essential to identify relaxer behavior, Maxwell–Wagner polarization, or interfacial effects. Materials like BaTiO_3_ and SrBi_2_Nb_2_O_9_ have shown such frequency dispersion and are used in capacitors and microwave devices [44,45]. Electrical conductivity in perovskites is often influenced by oxygen vacancies, carrier hopping, and polaronic transport. In Bi-containing perovskites, the mixed valence nature of bismuth can facilitate small polaron hopping, which manifests as thermally activated conduction. Studies on La_1−x_Sr_x_MnO_3_ and BiFeO_3_ have shown that the nature of charge carriers can be drastically modified by doping, sintering temperature, and synthesis route [46].

Magnetic characterization is equally important, especially for multifunctional materials. While BaBiO_3_ is typically non-magnetic, doping or deviation from stoichiometry can lead to the appearance of weak ferromagnetism or antiferromagnetism [47,48,49]. BiFeO_3_ is a classic example of a multiferroic perovskite that exhibits both ferroelectric and antiferromagnetic behavior at room temperature. The observation of magnetic ordering in unexpected systems like BiBaO_3_ may suggest defect-mediated magnetism, a phenomenon observed in many oxide systems [50]. In recent years, the demand for multifunctional materials that combine electric, dielectric, and magnetic properties has led to a surge in research on novel or modified perovskite compositions. Materials exhibiting more than one ferroic order are especially interesting for sensors, memory devices, and spintronic applications. Despite the wealth of information available on BaBiO_3_ and other bismuth-based perovskites, very limited work has been reported on BiBaO_3_ [51,52,53,54]. This suggests a significant research gap, and exploring this composition could contribute to the discovery of new structural phases and property combinations. Furthermore, no prior reports exist, to the best of our knowledge, on BiBaO_3_ synthesized via the sol–gel route, making this investigation both novel and potentially impactful.

In addition to the sol–gel method explored in this study, Bi-based perovskites such as BaBiO_3_ are commonly synthesized using solid-state reaction, hydrothermal, and combustion techniques [55]. However, these methods often require higher temperatures and may result in phase inhomogeneities, large grain sizes, or incomplete mixing of precursors. In contrast, the sol–gel method offers key advantages such as molecular-level mixing, lower crystallization temperatures, and improved control over particle morphology and stoichiometry [56]. These features contribute directly to enhanced dielectric response, reduced leakage, and more uniform grain boundary behavior. The present work highlights how the sol–gel route can yield BiBaO_3_ with improved microstructural uniformity and weak ferromagnetic response, offering potential benefits over more traditional synthesis approaches. The present study aims to synthesize BiBaO_3_ perovskite using the sol–gel method and perform a comprehensive investigation of its structural, microstructural, dielectric, electrical, and magnetic properties. Additionally, the results obtained will be compared with the existing literature on BaBiO_3_ and related perovskites to assess the influence of the synthesis method and compositional variation on the observed properties.

## 2. Synthesis and Characterization

### 2.1. Sample Preparation

The BiBaO_3_ perovskite material was synthesized using the sol–gel method, which is known for its ability to produce homogeneous and fine powders at low processing temperatures. High purity starting precursors were used, including bismuth nitrate pentahydrate [Bi(NO_3_)_3_·5H_2_O] and barium carbonate [BaCO_3_]. Initially, 20 mL of distilled water was used to dissolve the stoichiometric amount of Bi(NO_3_)_3_·5H_2_O. In a separate beaker, BaCO_3_ was dispersed in 40 mL of dilute nitric acid (HNO_3_) under continuous stirring. The acid facilitates the dissolution of BaCO_3_ by forming soluble Ba(NO_3_)_2_ and releasing CO_2_ gas. After full dissolution and clarification of the two solutions, they were mixed and stirred thoroughly. The mixed solution was maintained at approximately 70 °C for 5 h under continuous heating and stirring, during which a transparent solution formed. Once a uniform mixture is achieved, ethylene glycol and citric acid were added as complexing and gelling agents in a 1:1 molar ratio with respect to the total metal ions.

Ethylene glycol acts as a polymerization agent, while citric acid binds metal ions, ensuring a more homogeneous distribution. Stirring continued until a viscous and opaque gel formed, indicating the successful formation of a polymeric network containing the metal cations. The gel was then dried in an oven at 120 °C for 12 h to remove residual water and solvents, resulting in a dry foamy precursor. The dried gel was subjected to calcination in air at different temperatures of 500 °C, 600 °C, 700 °C, and 800 °C for 10 h each, to study the effect of temperature on phase formation and crystallinity. The resulting powders were then ground to obtain fine, homogeneous BiBaO_3_ powders for further characterization. To evaluate the sintering behavior and prepare the sample for electrical and magnetic measurements, pellets of the BiBaO_3_ powder were pressed using a uniaxial press and sintered at optimal temperatures identified through structural analysis.

After synthesis and calcination, the resulting BiBaO_3_ powders were divided into portions for various characterization techniques. For dielectric and electrical measurements, the powders were pressed into disk-shaped pellets and sintered at the optimal temperature. For magnetic measurements, a separate portion of the powder was used in loose form to evaluate the magnetic properties using SQUID magnetometry. Additional portions were allocated for structural and morphological analysis via XRD, SEM, and EDX. All measurements were performed on three independent synthesis batches to ensure reproducibility, and consistent trends were observed across all sample sets.

### 2.2. Sample Characterization

The structural characterization of the sample was conducted using several techniques. X-ray Diffraction was performed using an Empyrean diffractometer (Malvern Panalytical Ltd., Malvern, UK) (CuKα radiation, λ = 1.54060 Å) at 45 kV and 40 mA in the Bragg–Brentano prefocusing optics configuration. The step counting method was applied, with a step size of 0.02° and a time per step of 1 s, covering a 2θ angle range of 10° to 70°. Scanning electron microscopy and energy-dispersive X-ray spectroscopy measurements were performed using a Bruker Nano GmbH system, equipped with an XFlash 5010 silicon drift detector (Bruker, Billerica, MA, USA). The SEM analysis was conducted at a primary electron beam energy of 15 keV, providing detailed surface morphology. EDX was carried out in energy-dispersive mode using Esprit software version 2.3 (Bruker) for spectral acquisition and elemental mapping, with a take-off angle of 35° employed to optimize X-ray signal detection. A take-off angle of 35° was used during EDX analysis to optimize signal detection. To enhance the conductivity of the sample, a carbon coating was applied to its surface before observation. For electrical characterization, silver conducting paste was applied to the opposite sides of the pellets, and measurements were conducted in a helium atmosphere to reduce moisture and improve heat transfer. The electrical properties were analyzed using impedance spectroscopy (IS), with a frequency range of 100 Hz to 1 MHz and a temperature range of 200 to 380 K, using an Agilent 4294A precision impedance analyzer in the C_p_-R_p_ configuration. The complex permittivity (ε′, ε″) and loss tangent (tan δ) were calculated using the following equations [57,58]:

(1) $$\varepsilon^{\prime }=\frac{d}{A}\frac{C_{P}}{\varepsilon_{0}}$$

(2) $$\varepsilon^{\prime \prime }=\frac{d}{A}\;\frac{1}{\omega R_{P}\varepsilon_{0}}$$

(3) $$\tan\;\delta =\frac{\varepsilon^{\prime \prime }}{\varepsilon^{\prime }}$$

where C_p_ and R_p_ represent the measured capacitance and resistance, ω is the angular frequency, d is the sample thickness, A is the electrode area, and ε_0_ is the vacuum permittivity (8.8542 × 10^−12^ F/m). The magnetic properties of the sample were investigated using a Quantum Design MPMS3 superconducting quantum interference device (SQUID) magnetometer (Quantum Design, San Diego, CA, USA). Magnetization curves were measured at temperatures of 5 K, 300 K, and 380 K, with applied fields ranging from −70 kOe to 70 kOe. To correct sample geometry and offset effects, the geometry-independent moment correction (GIMC) method was applied. Additionally, temperature-dependent magnetic measurements were conducted using a Field-Cooled (FC) method, with a magnetic field of 1000 Oe, and the sample was heated from 5 to 380 K. Although gas adsorption measurements such as BET or Langmuir surface area and micropore volume analysis are often valuable for understanding porosity in sol–gel-derived materials, our characterization focused on detailed SEM imaging and EDX analysis across multiple calcination temperatures to assess microstructural evolution. These methods effectively revealed grain connectivity, surface morphology, and compositional uniformity, providing insights directly relevant to the dielectric and electrical performance discussed in this work. A broader surface area-dependent study is currently under consideration as part of future investigations aimed at correlating porosity with functional behavior in BiBaO_3_.

## 3. Results and Discussion

### 3.1. X-Ray Diffraction Analysis

**Figure 1:**
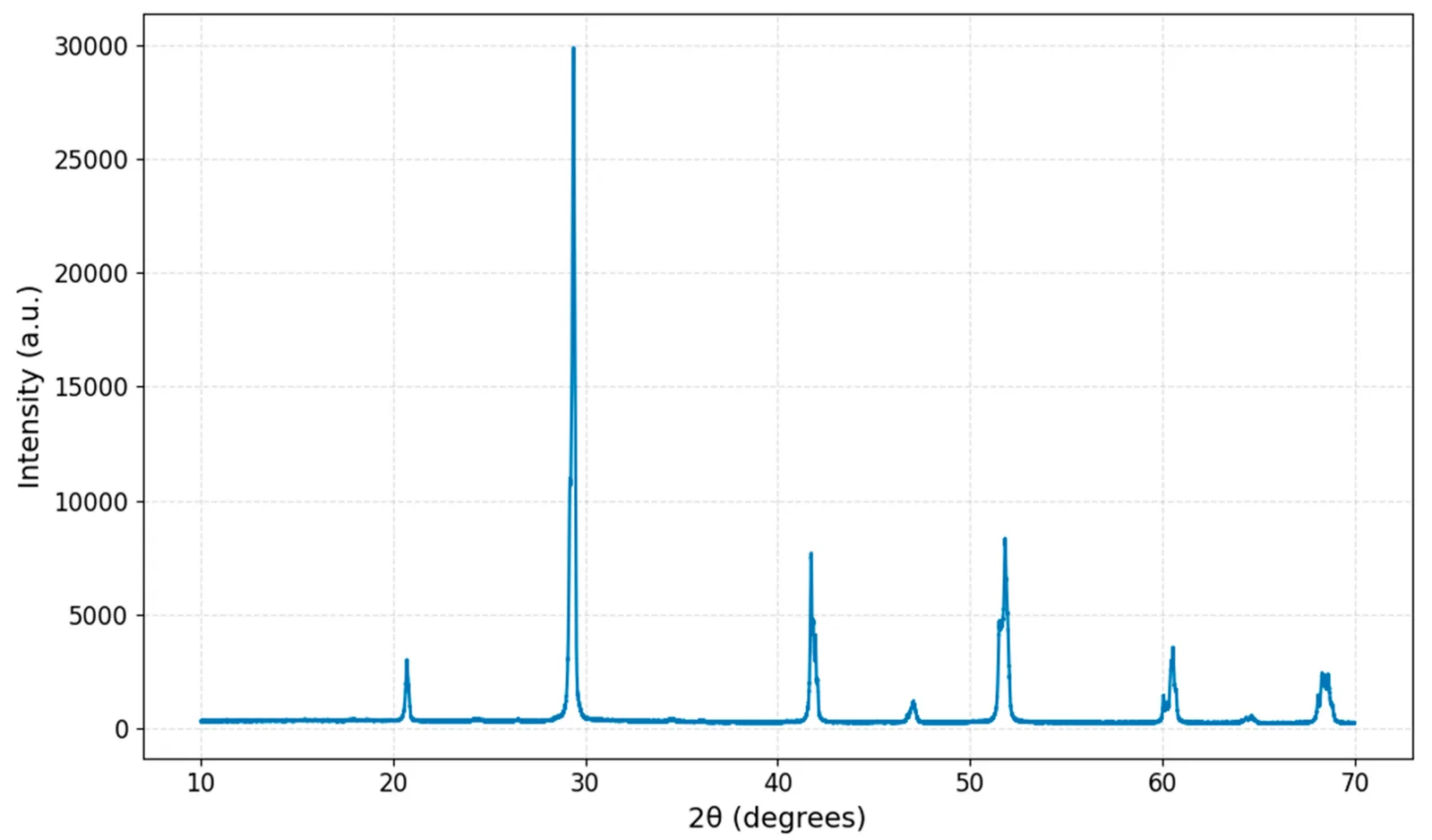
X-ray diffraction (XRD) pattern of BiBaO_3_ synthesized at 700 °C, exhibiting sharp and well-defined peaks indicative of high crystallinity.

The XRD pattern of the synthesized BiBaO_3_ sample exhibits sharp and well-defined peaks, indicating a high degree of crystallinity. The prominent diffraction peaks are observed at 2θ values of approximately 22.3°, 27.6°, 31.7°, 40.1°, 46.5°, 52.6°, 57.3°, 63.0°, and 67.1°, with the most intense peak near 31.7°, suggesting a dominant crystallographic orientation. The XRD pattern of the prepared sample, as shown in Figure 1, exhibits well-defined peaks. The absence of additional peaks suggests phase purity, with no detectable secondary phases. To estimate the average crystallite size (D) of the sample, the Scherrer equation was employed [59]:

(4) $$D_{\mathrm{XRD}}=\frac{K.\lambda }{\beta .cos\theta }$$

where D_XRD_ is the average crystallite size, K is the shape factor (typically 0.9), λ is the X-ray wavelength (1.5406 Å for Cu Kα radiation), β is the full width at half maximum (FWHM) of the peak in radians, and θ is the Bragg angle (half of the 2θ value).

The FWHM values were corrected for instrumental broadening using the following relation [60]:

(5) $$\beta \mathrm{corrected}=\sqrt{{\beta_{measured}}^{2}-{\beta_{instrumental}}^{2}}$$

**Figure 2:**
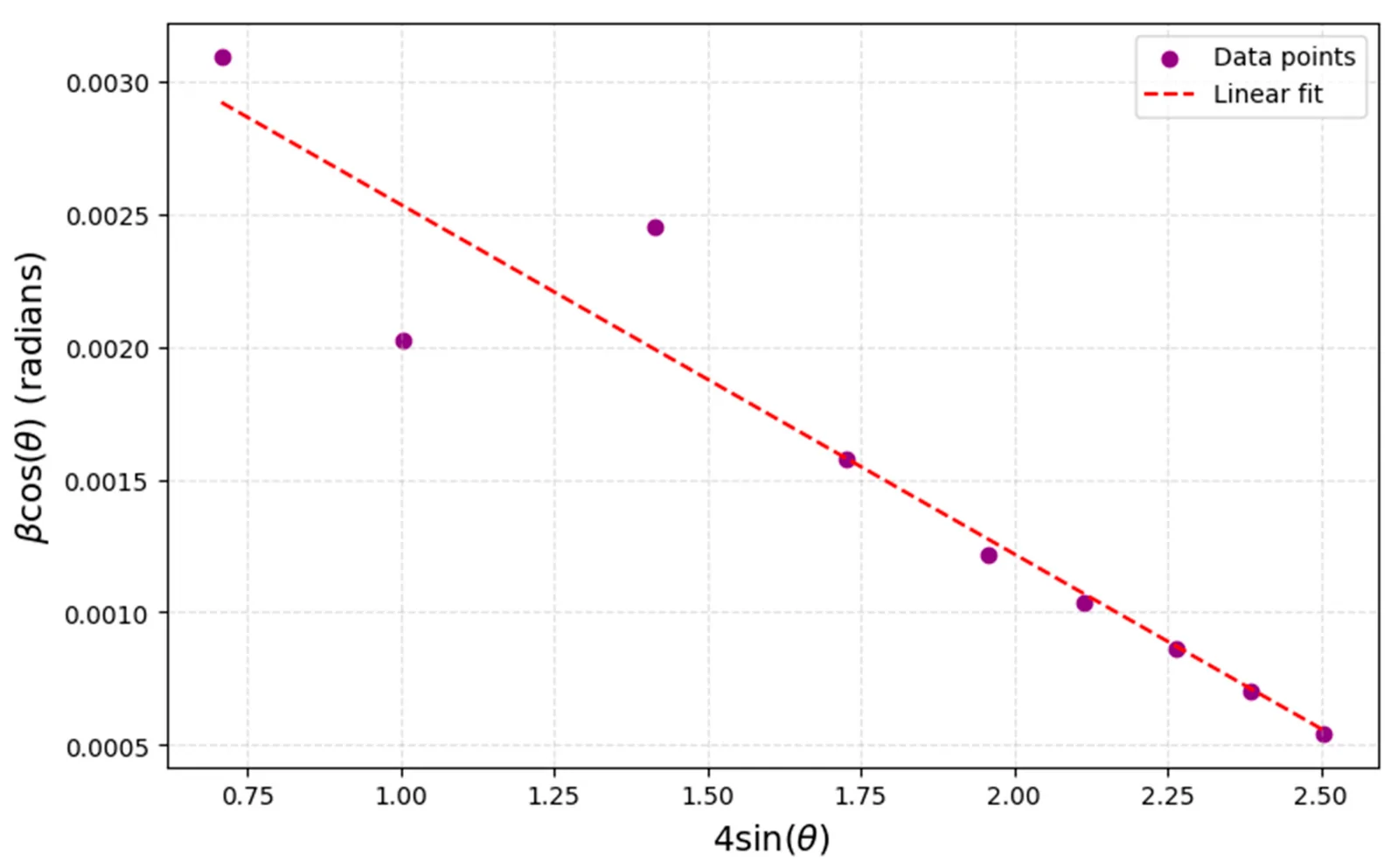
Williamson–Hall plot for BiBaO_3_ sample.

Assuming negligible instrumental broadening, the crystallite sizes for the prominent peaks were calculated as follows (Table 1):

These results indicate that the crystallite sizes range from approximately 45.9 nm to 125.8 nm, with larger crystallites corresponding to higher-angle reflections. To further analyze the microstructural properties, the Williamson–Hall (W-H) method applied. This method considers both size-induced and strain-induced broadening of XRD peaks. The W-H equation is as follows [61]:

(6) $$\beta \mathrm{Cos}\;(\theta )=\frac{K\lambda }{D_{WH}}+4\;\varepsilon \sin\;(\theta )$$

where β is the FWHM in radians, θ is the Bragg angle, and ε is the Var epsilon is the microstrain. By plotting β cosθ against 4 sinθ, a linear fit yields the crystallite size from the intercept and the microstrain from the slope. Figure 2 illustrates the Williamson–Hall plot for the BiBaO_3_ sample. The linear fit to the data points yields a crystallite size of approximately 65.3 nm and a microstrain of 0.0012 (or 0.12%). These values suggest that the sample possesses relatively large crystalline strain with minimal lattice strain, indicative of a well-ordered crystalline structure.

The crystallite sizes of the BiBaO_3_ sample were analyzed using both the Scherrer equation and the Williamson–Hall method. The Scherrer equation estimated the crystallite size to be in the range of 45.9 to 125.8 nm, whereas the Williamson–Hall method provided an average size of 65.3 nm. Unlike the Scherrer equation, which considers only size-induced broadening of diffraction peaks, the Williamson–Hall approach considers both crystallite size and microstrain effects, offering a more comprehensive understanding of the sample’s microstructure. The slight difference between the two results highlights the presence of microstrain in the material; however, the low microstrain value indicates minimal lattice distortions. This low strain is beneficial for the material’s structural stability and potential applications. The consistency between the crystallite sizes obtained from both methods further confirms the reliability of the measurements and demonstrates the effectiveness of the Williamson–Hall analysis in capturing the material’s microstructural properties.

To determine the crystal structure and refine the lattice parameters of the BiBaO_3_ sample, Rietveld refinement was performed using the FullProf Suite. This refinement involved fitting the entire XRD pattern to a structural model, allowing precise extraction of crystallographic information. The results reveal that BiBaO_3_ crystallizes in a monoclinic structure with the space group P2_1/n_. The refined lattice parameters were found to be a = 6.183 Å, b = 6.137 Å, c = 8.648 Å, and β = 90.0°, confirming the monoclinic symmetry of the material. Based on these parameters, interatomic distances were calculated, yielding Ba–O and Bi–O distances of 2.75 Å and 2.60 Å, respectively, which align well with the expected coordination environments in BiBaO_3_. These structural parameters are consistent with previously reported values for BiBaO_3_ synthesized under similar conditions, such as at temperatures around 700 °C, reinforcing the reliability of the refinement and confirming the phase identification.

Studies on the related compound BaBiO_3_ have reported similar structural characteristics, including monoclinic symmetry and comparable lattice parameters, reinforcing the reliability of our results. For instance, Korotin et al. [62] investigated electronic correlations and crystal structure distortions in BaBiO_3_ and successfully reproduced the monoclinic phase, emphasizing the role of breathing distortions and octahedral tilting in stabilizing this structure. Their theoretically derived lattice parameters closely match our experimental findings, further supporting the validity of our structural model. Regarding interatomic distances, our calculated Ba–O and Bi–O bond lengths of 2.75 Å and 2.60 Å, respectively, are in good agreement with values reported in the Materials Project database, which lists Ba–O distances ranging from 2.60 to 2.93 Å and Bi–O distances from 2.24 to 2.66 Å for BaBiO_3_. This agreement further substantiates the accuracy of our structural analysis. Additionally, Plumb et al. [63] conducted angle-resolved photoemission spectroscopy on BaBiO_3_ thin films and observed Brillouin zone folding consistent with BiO_6_ breathing distortions, supporting the existence of a monoclinic structure. Their findings corroborate ours, highlighting the structural stability of BiBaO_3_ in the monoclinic phase.

### 3.2. Morphological Study

**Figure 3:**
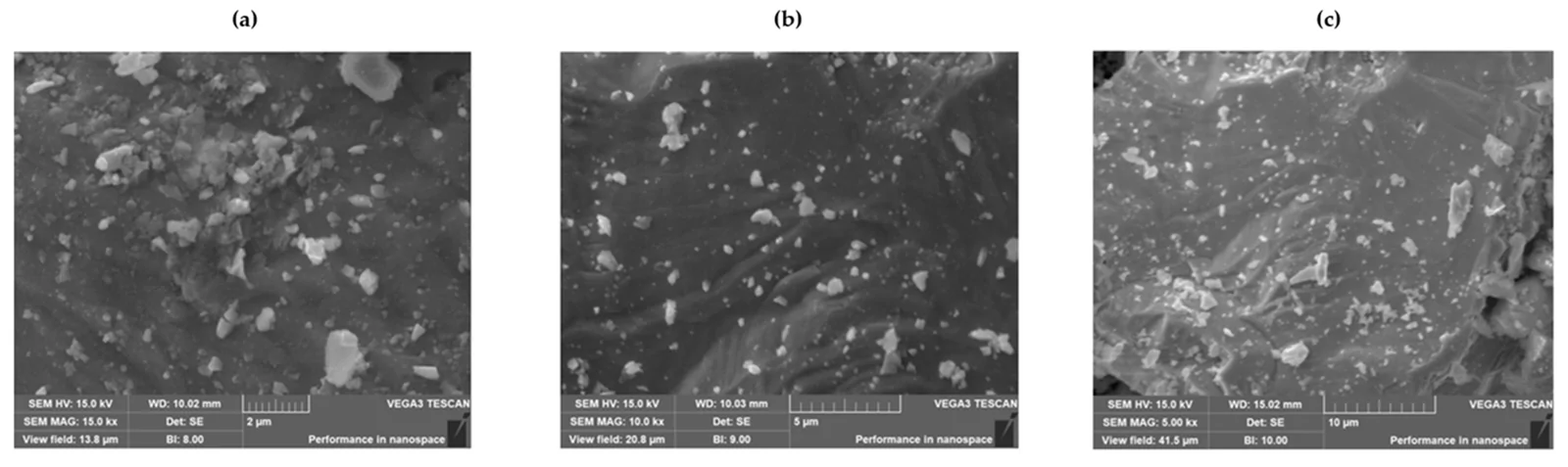
Scanning electron microscopy (SEM) images of the BiBaO_3_ ceramic, showing (**a**) a microstructure captured at 15,000× magnification with a 2 µm scale bar, displaying agglomerated and irregular grains with high porosity; (**b**) an image taken at 10,000× magnification with a 5 µm scale bar, showing smoother surfaces and moderate grain growth with partial interconnection; and (**c**) an image at 5000× magnification with a 10 µm scale bar, illustrating a denser grain structure with more continuous boundaries and reduced porosity.

The surface morphology of the prepared BiBaO_3_ ceramic was examined using scanning electron microscopy, and the elemental composition was analyzed through energy-dispersive X-ray spectroscopy. As shown in Figure 3, SEM images of the sample synthesized at 700 °C reveal significant microstructural evolution characteristic of sol–gel-derived oxide ceramics. In Figure 3a (scale: 2 μm), the microstructure appears heterogeneous, with a high degree of grain agglomeration and irregularly shaped particles. The surface shows pronounced porosity and uneven grain distribution, indicating incomplete densification. These features are commonly attributed to the evolution of gases during the thermal decomposition of organic precursors in sol–gel synthesis. Figure 3b (scale: 5 μm), the ceramic surface becomes relatively smoother, and some degree of grain growth is evident. The porosity is less prominent, and the grain contacts are more developed, suggesting that the sintering process has initiated neck formation between grains and Figure 3c (scale: 10 μm) shows a more interconnected grain structure with reduced porosity and clearer grain boundaries. Larger grain clusters are visible, implying enhanced grain coalescence and densification at this stage. This interconnected network is favorable for improving the electrical conductivity and dielectric response of perovskite-based ceramics such as BiBaO_3_.

**Figure 4:**
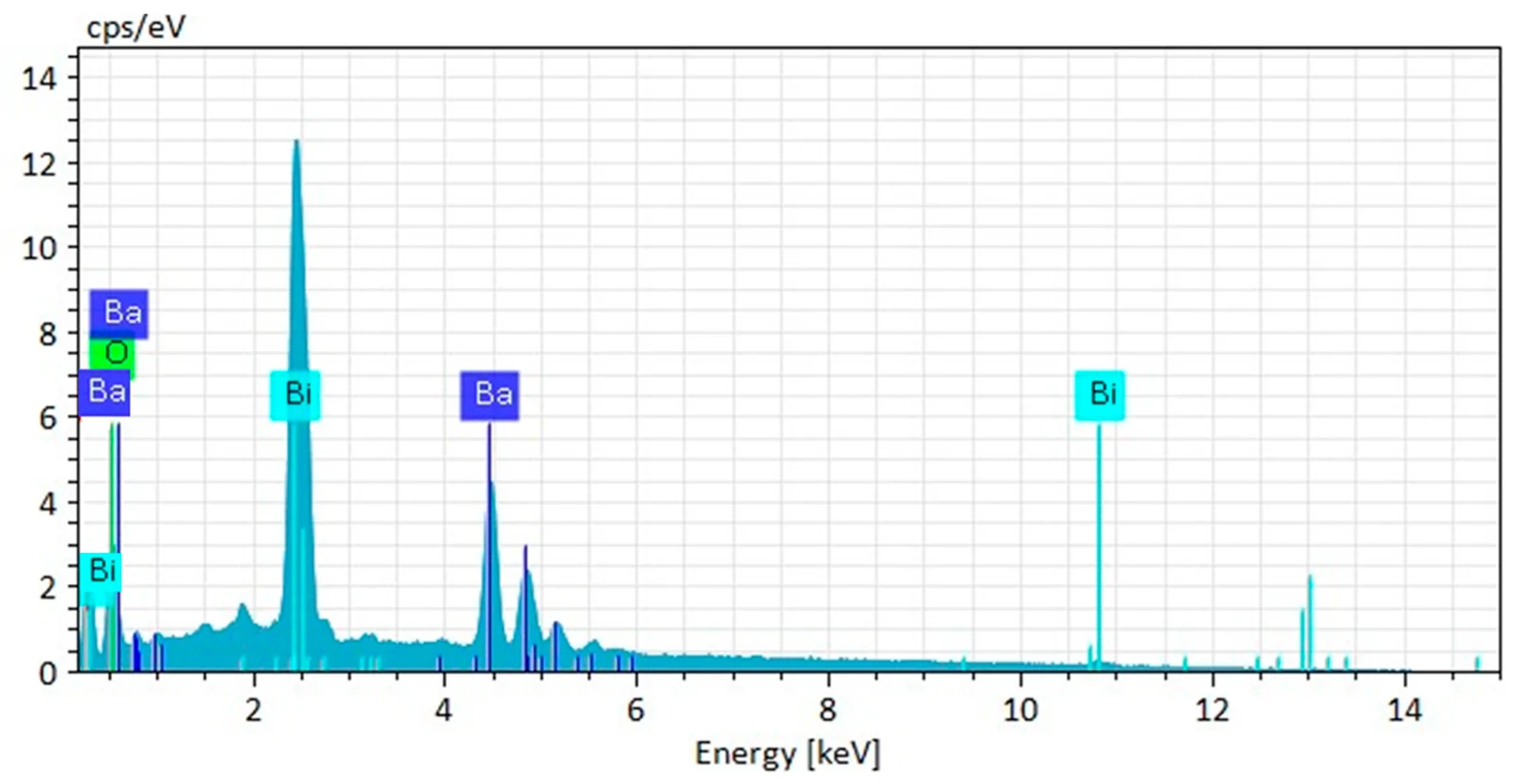
Energy-dispersive X-ray spectrum of the BiBaO_3_ sample confirming the elemental composition, with prominent peaks corresponding to bismuth (Bi), barium (Ba), and oxygen (O), consistent with the expected stoichiometry of the compound.

To further investigate the chemical composition, EDX analysis was conducted on the same area, and the resulting spectrum is presented in Figure 4. The spectrum confirms the presence of the main constituent elements, bismuth (Bi), barium (Ba), and oxygen (O), in proportions that closely align with the expected stoichiometry of BiBaO_3_. The semi-quantitative data shows a predominant concentration of bismuth (50.70 wt%) and barium (34.57 wt%), with oxygen comprising 8.98 wt%. A small amount of carbon (5.74 wt%) was also detected, which is attributed to either residual organics from the synthesis or the carbon tape used for mounting the sample. The absence of any impurity peaks in the EDX spectrum indicates the successful formation of a chemically pure single-phase material. The relatively high bismuth content supports the structural distortion observed in XRD results, suggesting the incorporation of Bi^3+^ ions into the perovskite lattice and possibly contributing to oxygen vacancy formation. These morphological and compositional findings not only confirm the successful synthesis of BiBaO_3_ but also provide insight into how the microstructure may influence the sample’s functional properties, such as its dielectric and magnetic behaviors.

The combination of SEM and EDX analysis provides a comprehensive view of the sample’s morphology and elemental composition, validating the effectiveness of the synthesis method and offering insights into the structural integrity and chemical homogeneity of the material.

### 3.3. Dielectric Properties

**Figure 5:**
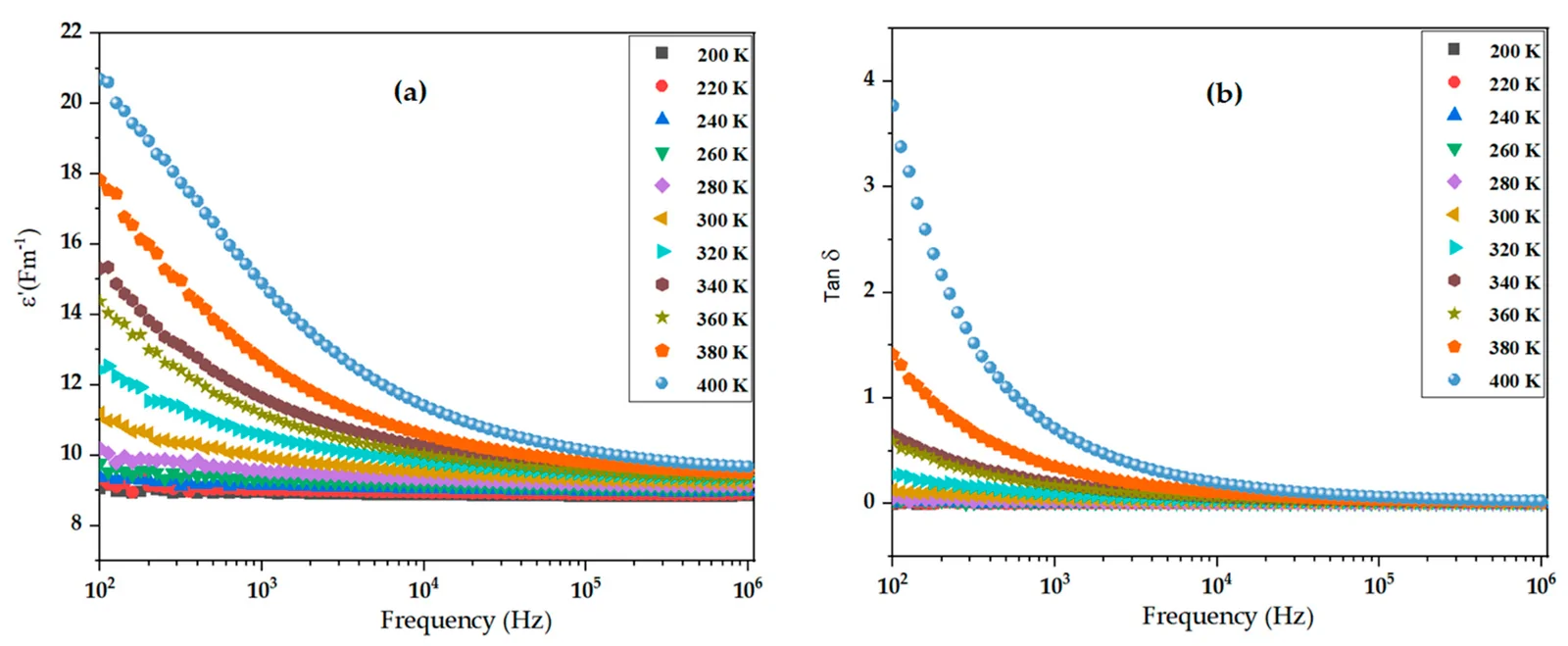
(**a**) Frequency and temperature dependence of the real part of dielectric permittivity (ε′) of BiBaO_3_. (**b**) Frequency and temperature dependence of dielectric loss tangent (tan δ) of BiBaO_3_.

The dielectric properties of the synthesized material were investigated over a wide frequency range (100 Hz to 1 MHz) and temperatures between 200 K and 400 K to understand its polarization mechanisms and energy dissipation behavior. Figure 5a illustrates the real part of the dielectric permittivity (ε′), and Figure 5b presents the dielectric loss tangent (tan δ) under these varying conditions. As shown in Figure 5a, ε′ exhibits a pronounced frequency-dependent dispersion across all temperatures. At low frequencies (~100 Hz), ε′ reaches its maximum value, especially at higher temperatures. As the frequency increases toward 1 MHz, the permittivity steadily decreases, showing typical relaxation behavior. Moreover, ε′ decreases with increasing frequency due to limited dipole mobility and the suppression of interfacial polarization and it increases with temperature, especially at lower frequencies, indicating thermally assisted polarization processes.

This trend can be attributed to the Maxwell–Wagner interfacial polarization mechanism, which dominates at low frequencies due to the presence of grain boundaries and interfaces within the polycrystalline material [64]. At lower temperatures (200 K), the ε′ values are relatively low across all frequencies, while at higher temperatures (400 K), the values increase significantly at low frequencies, indicating thermally activated polarization processes. This enhancement with temperature suggests the involvement of space charge accumulation at interfaces that become more mobile at elevated temperatures. The sharp decrease in ε′ with frequency at all temperatures suggests that dipolar or interfacial polarization mechanisms are strongly suppressed as the external field oscillates faster than the charge carriers or dipoles can respond [65].

Figure 5b shows the dielectric loss tangent (tan δ) as a function of frequency and temperature. Notably, tan δ remains relatively low across the entire frequency range, especially at higher frequencies. At low frequencies (~100 Hz) and higher temperatures (400 K), a small increase in tan δ is observed, likely due to enhanced conduction losses and charge carrier mobility. The low values of tan δ (below 0.1) even at elevated temperatures highlight the low dielectric loss behavior of the material, which is desirable for high-frequency applications such as capacitors, where minimal energy dissipation is critical. Also, tan δ slightly decreases with increasing frequency for all temperature conditions, which is consistent with reduced hopping conduction and energy loss as the field oscillates faster than the response time of charge carriers [66]. It remains low overall, showing only a slight increase at low frequencies and high temperatures, confirming the material’s low-loss dielectric nature. These behaviors reflect a typical non-Debye-type dielectric relaxation, associated with complex microstructural features and space charge dynamics. The stability of both ε′ and tan δ at high frequencies indicates the potential of the material for use in miniaturized electronic devices and high-frequency capacitor applications.

### 3.4. Electrical Conductivity and Modulus Analysis

**Figure 6:**
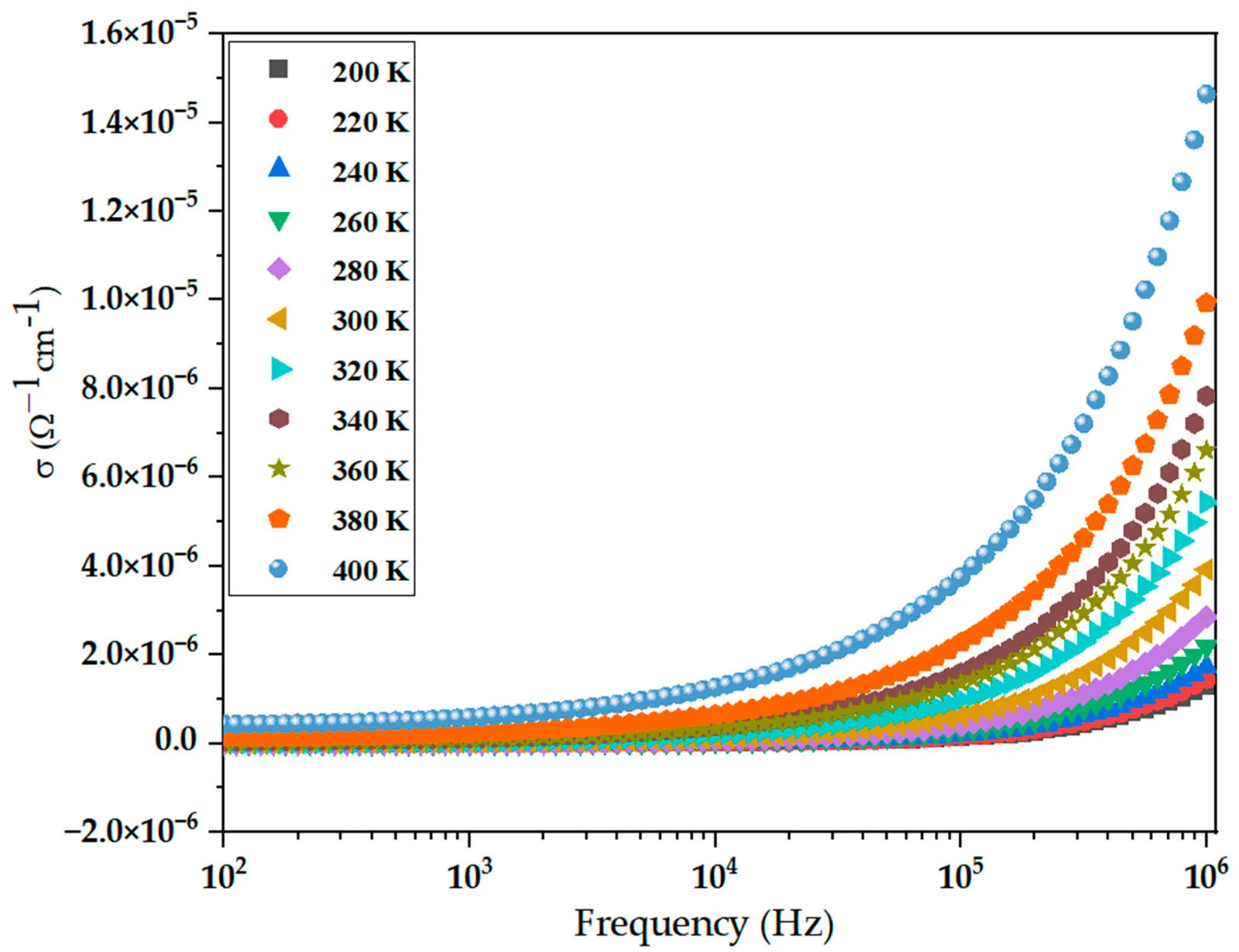
Frequency dependence of AC conductivity (σ_ac_) of BiBaO_3_ at various temperatures.

The electrical behavior of the BiBaO_3_ perovskite was thoroughly investigated by analyzing its AC conductivity and complex electric modulus over a frequency range from 100 Hz to 100 kHz and a temperature range of 200 K to 400 K. This specific temperature interval was selected to probe both low-temperature charge localization and high-temperature thermal activation effects. The evolution of electrical response with temperature reveals a rich transport mechanism involving both grain and grain boundary contributions. The variation in AC conductivity (σ_ac_) with frequency and temperature is depicted in Figure 6. At lower frequencies, σ_ac_ remains relatively constant, corresponding to DC-like behavior where mobile charge carriers contribute to long-range conduction. As the frequency increases, σ_ac_ increases significantly due to the short-range hopping of localized charge carriers. This behavior conforms to Jonscher’s universal power law [67,68]:

(7) $$\sigma_{Ac}\left(\omega \right)=\sigma_{dc}+A\omega^{s}$$

where $\sigma_{dc}$ is the DC conductivity, A is a temperature-dependent pre-factor, ω = 2πf is the angular frequency, and s is an exponent (0 < s < 1) that characterizes the conduction mechanism. The value of s generally decreases with increasing temperature, indicating a thermally activated hopping process where carriers gain sufficient energy to overcome potential barriers between localized states.

**Figure 7:**
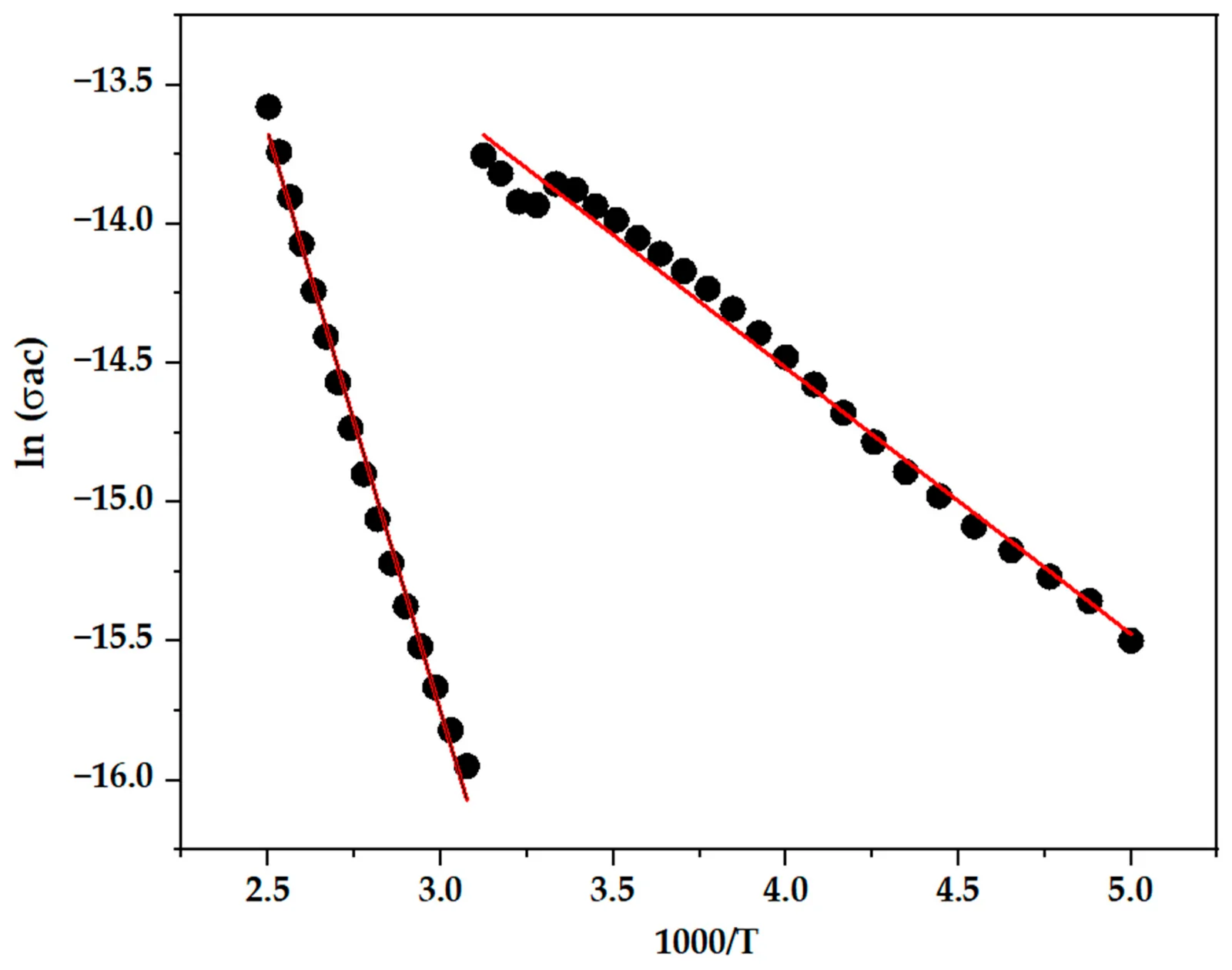
Arrhenius plot of ln(σ_ac_) vs. 1000/T for BiBaO_3_: extraction of activation energies.

To further elucidate the conduction mechanism, the temperature dependence of σ_ac_ was analyzed using an Arrhenius plot, as shown in Figure 7. This plot, constructed by graphing ln (σ_ac_) versus 1000/T, allows the extraction of energy activation (E_a_) for charge transport. The data reveal two distinct linear regions, each corresponding to a different thermal regime. The relationship follows the Arrhenius equation [69,70,71]:

(8) $$\ln\;(\sigma_{\mathrm{ac}})=\ln\;(\sigma_{0})-\frac{-E_{a}}{K_{B}T}$$

where σ_0_ is the pre-exponential factor, E_a_ is the activation energy, K_B_ is Boltzmann’s constant, and T is the absolute temperature. Using linear fitting in Origin Pro 2025 software, the slopes of these two regions yield activation energy values of approximately 0.36 eV for the high-temperature regime and 0.18 eV for the low-temperature regime. The presence of two activation energies strongly suggests two predominant conduction processes: the lower activation energy is typically associated with grain boundary conduction or short-range hopping, while the higher energy corresponds to long-range bulk conduction within grains.

This dual activation behavior reflects the microstructural heterogeneity of the BiBaO_3_ material. Grain boundaries generally exhibit higher resistivity and dominate conduction at lower temperatures due to their barrier potential. As temperature increases, the grains become more conductive due to enhanced carrier mobility, and bulk conduction becomes the primary mechanism [72]. To deepen the understanding of dielectric and relaxation phenomena in BiBaO_3_, the electric modulus formalism was applied. The complex electric modulus is defined as follows [73]:

(9) $$M^{*}\;(\omega )=\frac{1}{\varepsilon^{*}\left(\omega \right)}=M^{\prime }+j\;M^{\prime \prime }$$

The equation for the modulus technique’s general relation was derived and is presented as follows [74]:

(10) $$M^{\prime }=A\;[\frac{{\left(\omega RC\right)}^{2}}{{1+\left(\omega RC\right)}^{2}}]=A\;[\frac{{\tau^{2}\omega }^{2}}{1+{\tau^{2}\omega }^{2}}]$$

(11) $$M^{\prime \prime }=A\;[\frac{\left(\omega RC\right)}{{1+\left(\omega RC\right)}^{2}}]=A\;[\frac{\omega \tau }{1+{\tau^{2}\omega }^{2}}]$$

**Figure 8:**
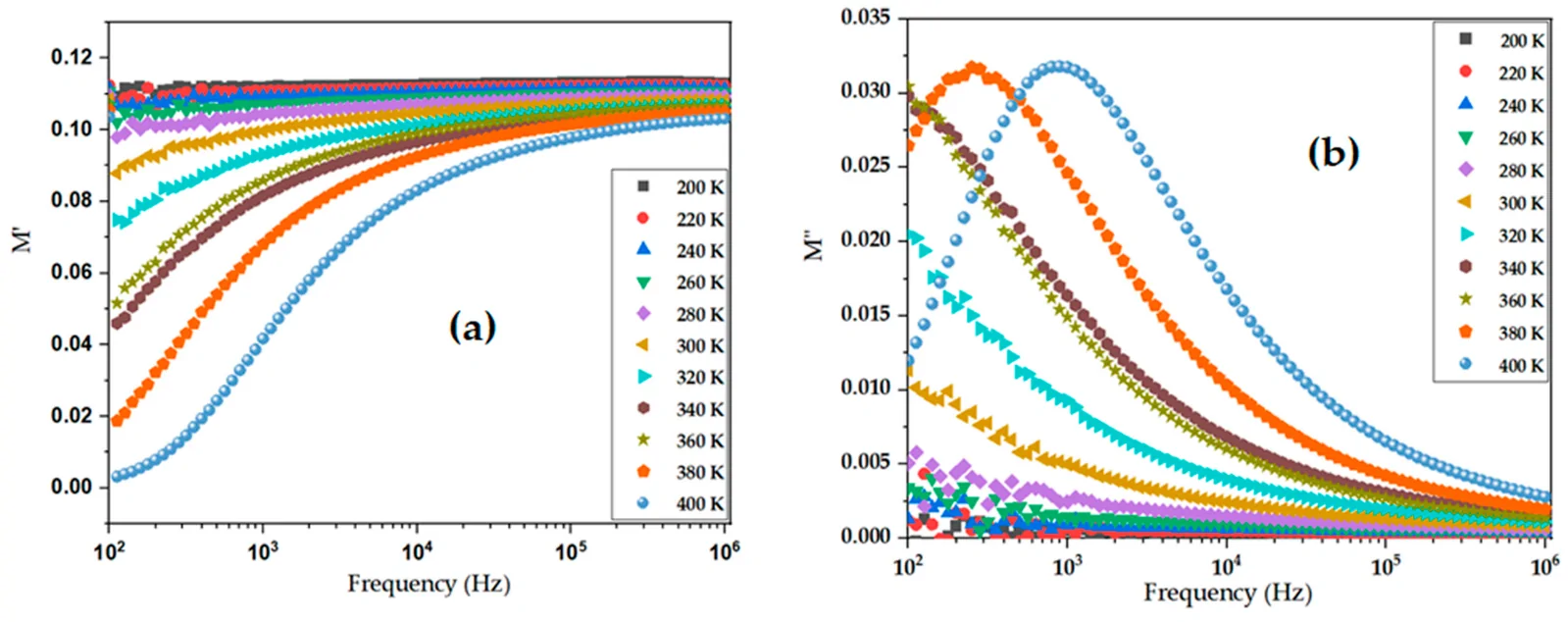
Frequency dependence of electric modulus components of BiBaO_3_ at various temperatures: (**a**) real part (M′), showing dispersion with frequency; (**b**) imaginary part (M″), indicating thermally activated dielectric relaxation behavior.

In the equation, A represents the ratio of capacitance (C) in vacuum to dielectric (Co/C), R stands for resistance, ω denotes the angular frequency of the applied AC field, ε∗(ω) is the complex dielectric permittivity, and M′ and M″ are the real and imaginary parts of the electric modulus, respectively. The real part of the electric modulus, M′, as presented in Figure 8a, provides critical insights into the long-range conductivity and dielectric behavior of BiBaO_3_. At low frequencies, M′ approaches zero, which suggests that the material allows for significant long-range charge transport without strong impedance from interfacial polarization (i.e., electrode effects) [75]. This behavior confirms that the response in this region is dominated by DC conductivity and indicates the existence of mobile charge carriers that traverse larger distances. As the frequency increases, M′ begins to rise, showing a dispersion behavior. This increase is linked to the onset of relaxation phenomena, where the electric field begins to interact with more localized charge dynamics, such as trapped charges or hopping between localized states [76]. The shift in the M′ curve to higher frequencies with increasing temperature suggests a thermally activated process, where increased thermal energy allows charges to respond more rapidly to the alternating field [77]. Such behavior is characteristic of dielectric relaxation due to localized charge motion becoming more significant at elevated temperatures.

In Figure 8b, the imaginary part of the modulus, M″, is plotted against frequency and shows well-defined peaks for each temperature. These peaks represent the characteristic relaxation frequency f_max_ of the material, where the imaginary part reaches a maximum, indicating the most efficient energy loss from the system due to relaxation processes. Notably, the M″ peak shifts towards higher frequencies with increasing temperature, a hallmark of thermally activated relaxation mechanisms [78]. This shift indicates that as temperature increases, the relaxation time τ of the system decreases, allowing the system to relax faster in response to the applied field. The relationship between the relaxation time τ and peak frequency f_max_ is given by the following equation:

(12) $$\tau =\frac{1}{2\pi f_{max}}$$

Furthermore, the temperature dependence of τ can be described by an Arrhenius-type equation, which allows the extraction of the activation energy associated with the relaxation process:

(13) $$\ln\;(\tau )=\ln\;(\tau_{0})+\frac{-E_{a}}{K_{B}T}$$

where $\tau_{0}$ is the pre-exponential factor, E_a_ is the activation energy for dielectric relaxation, K_B_ is the Boltzmann constant, and T is the absolute temperature in Kelvin. This model explains how increased thermal energy facilitates faster charge mobility and shorter relaxation times. The well-defined and thermally shifting peaks in M′′ support the existence of localized ionic or polaronic hopping mechanisms, particularly relevant in complex perovskite structures like BiBaO_3_. The analysis of M′ and M′′ provides robust evidence for the coexistence of long-range conductivity and localized relaxation phenomena in BiBaO_3_. These behaviors, together with the dual activation energies observed in the conductivity data, emphasize the significant role of both grain and grain boundary processes in shaping the dielectric and conductive properties of this material.

### 3.5. Impedance Spectroscopy Analysis

**Figure 9:**
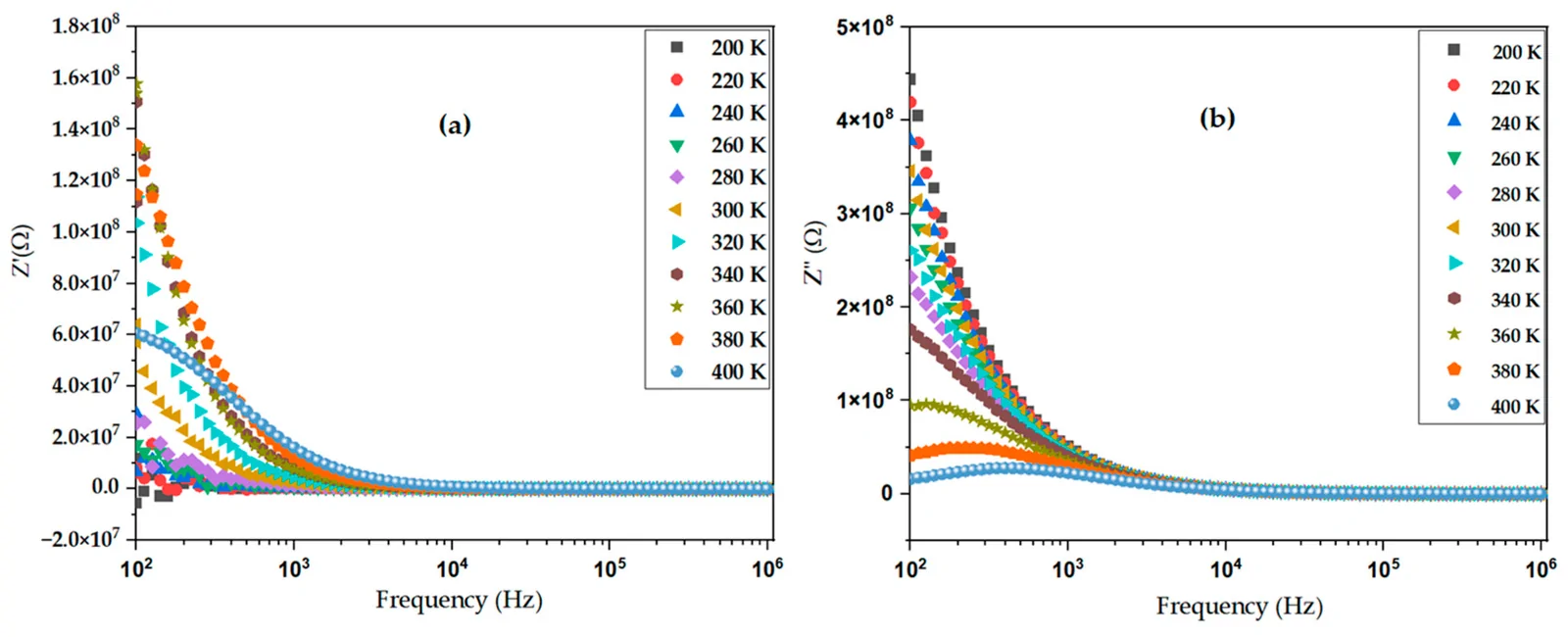
Frequency dependence of impedance spectroscopy of BiBaO_3_ at various temperatures: (**a**) real part (Z′), showing frequency-dependent dispersion; (**b**) imaginary part (Z).

Impedance spectroscopy is a powerful technique to investigate electrical properties and charge transport mechanisms in perovskite materials. In this study, the impedance response of the BiBaO_3_ sample was analyzed over a range of frequencies, with results presented in Figure 9a,b, showing the real (Z′) and imaginary (Z″) parts of the impedance, respectively. Figure 9a illustrates the frequency dependence of the real part of impedance. At low frequencies, a higher Z′ value is observed, indicating a dominant resistive behavior attributed to grain boundary and electrode effects [79]. As the frequency increases, Z′ decreases, demonstrating enhanced charge carrier mobility within the material’s bulk. This trend suggests the presence of a frequency-dependent conduction mechanism typically seen in perovskite oxides [80].

Figure 9b presents the imaginary part of impedance, which exhibits a peak corresponding to a relaxation process in the material. The peak position shifts toward higher frequencies with increasing temperature, indicating thermally activated relaxation phenomena [81]. This peak corresponds to the characteristic relaxation time of charge carriers or dipolar species in the BiBaO_3_ lattice. Together, these impedance spectra provide insights into the electrical conduction and polarization processes in the prepared sample. The semicircular arcs typically reflect the bulk and grain boundary contributions to impedance, allowing for equivalent circuit modeling to extract resistance and capacitance parameters. Overall, the impedance spectroscopy analysis confirms that the electrical behavior of BiBaO_3_ is governed by both bulk and interfacial effects, crucial for optimizing its performance in electronic and energy devices [82].

### 3.6. Magnetic Properties

**Figure 10:**
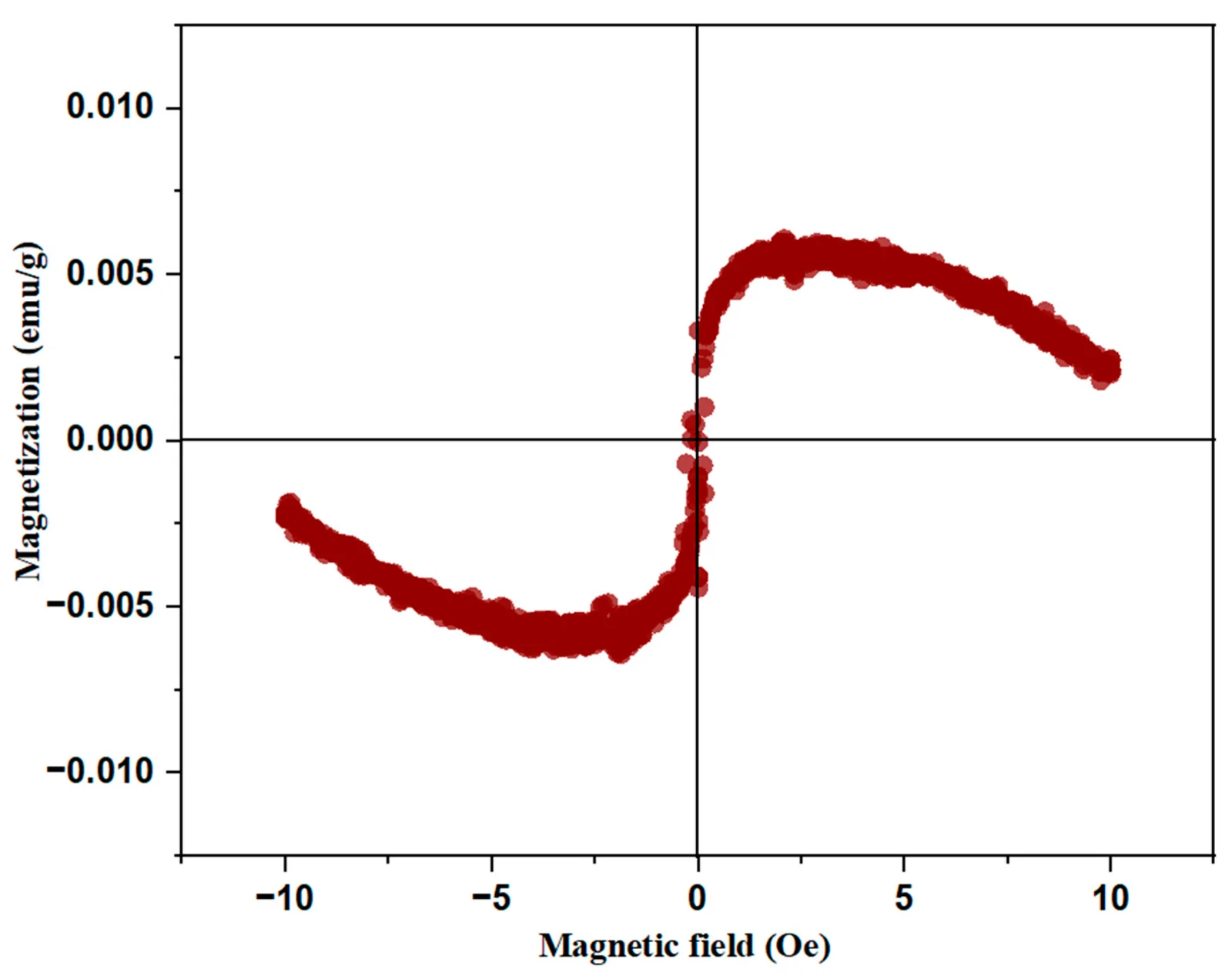
Magnetic hysteresis loop of BiBaO_3_ at room temperature.

The magnetic behavior of BiBaO_3_ was investigated at room temperature using vibrating sample magnetometry, with the corresponding hysteresis loop shown in Figure 10. The loop exhibits a narrow, symmetric S-shaped curve centered near the origin, indicative of weak ferromagnetic or soft magnetic characteristics. Quantitative analysis of the curve yields a saturation magnetization (M_s_) of approximately 0.0058 emu/g, a remanent magnetization (M_r_) near 0.0004 emu/g, and an extremely low coercive field (H_c_) around 0.7 Oe. These values clearly demonstrate that BiBaO_3_ exhibits minimal magnetic ordering with very low hysteresis losses, typical of materials that possess soft magnetic behavior or weak ferromagnetism rather than strong or hard ferromagnetic ordering. The origin of this magnetic response in BiBaO_3_ is intriguing, given that both Bi^3+^ and Ba^2+^ ions are nominally non-magnetic [83]. However, subtle effects arising from the complex crystal chemistry of perovskite oxides can lead to emergent magnetism. One crucial factor is the possibility of mixed valence states such as Bi^3+^/Bi^4+^ and Ba^2+^/Ba^3+^, which may develop due to oxygen vacancies or synthesis conditions that alter stoichiometry. These mixed valence ions enable electron hopping via double exchange mechanisms, which facilitate the alignment of neighboring spins and generate ferromagnetic coupling despite the absence of traditional magnetic ions [84,85]. In addition, super exchange interactions mediated through oxygen ions may contribute further complexity to the magnetic structure, with the nature of coupling (ferromagnetic or antiferromagnetic) depending strongly on local bonding geometries and orbital overlaps.

Moreover, the presence of the stereochemical active 6 s^2^ lone pair on Bi^3+^ ions introduce lattice distortions that disrupt the ideal symmetry of the perovskite framework. These distortions can cause spin canting, where the magnetic moments tilt away from perfect antiparallel alignment, resulting in a small net magnetization and weak ferromagnetism. Oxygen vacancies exacerbate this effect by creating localized states and modifying the electronic environment, which enhances the likelihood of unpaired spins and mixed valence formations. These factors collectively give rise to defect-driven magnetism in the otherwise non-magnetic BiBaO_3_ lattice. The nearly linear increase in magnetization at higher applied fields and the lack of a well-defined saturation plateau suggest that the material does not achieve full magnetic saturation, likely due to competing interactions such as antiferromagnetic coupling coexisting with weak ferromagnetic domains [86].

Taken together, the magnetic properties of BiBaO_3_ highlight the subtle interplay between electronic structure, lattice distortions, and defect chemistry in determining its magnetic ground state. The weak ferromagnetic signature observed, despite the absence of conventional magnetic ions, underscores the potential of the sample as a multifunctional perovskite oxide where magnetism can be tuned through controlled doping, defect engineering, or external stimuli. Such tunability opens prospects for applications in spintronics, sensors, and magnetoelectric devices, where a controlled weak magnetic response is desirable. Further investigations, including temperature-dependent magnetic measurements and theoretical modeling, will be essential to fully elucidate the microscopic mechanisms governing the emergent magnetism in the prepared sample. In this study, magnetic characterization was carried out at room temperature, where BiBaO_3_ exhibited clear signatures of weak ferromagnetic behavior. These findings are significant, as they confirm the presence of defect-driven magnetism in the material, despite the absence of conventional magnetic ions. The results are consistent with structural distortions and mixed valence effects that emerge during sol–gel synthesis. While our primary emphasis was on analyzing the dielectric, electrical, and impedance properties over a broad temperature range (200–400 K), the magnetic measurements at ambient conditions provided valuable initial insight into the magnetic nature of BiBaO_3_. In future work, we aim to extend the magnetic analysis to cover a wider temperature range and incorporate theoretical modeling approaches such as the Jiles–Atherton model. Such studies will further enhance understanding of the material’s magnetic domain dynamics and magneto-thermal behavior, building upon the solid foundation established in the present work.

Beyond the mechanisms previously discussed, it is worth noting that weak ferromagnetic signatures in materials like BiBaO_3_ may also stem from nanoscale phase inhomogeneities or interface effects between structurally distorted regions. In perovskites synthesized via sol–gel routes, the local strain fields, precursor reactivity, and calcination kinetics can result in subtle spatial variations that are not easily detected by bulk structural analysis yet have a strong influence on magnetic behavior. Techniques such as magnetic force microscopy (MFM) or Mössbauer spectroscopy could provide further insight into domain-level magnetic features and hyperfine interactions. Additionally, the presence of a low-field magnetic response may offer functionality in magnetic sensing or low-power spintronic applications where controlled weak magnetism is advantageous. These directions warrant future exploration to fully establish the link among synthesis conditions, local structure, and functional magnetic behavior in BiBaO_3_ and related systems.

## 4. Conclusions

In this study, BiBaO_3_ perovskite was successfully synthesized via the sol–gel method and subjected to a detailed investigation of its structural, electrical, dielectric, and magnetic properties. The structural characterization confirmed the formation of a single-phase perovskite structure with high crystalline, as evidenced by X-ray diffraction. SEM analysis further revealed a uniform microstructure, while EDX validated the stoichiometric composition of the synthesized material. Dielectric and impedance spectroscopy revealed significant frequency and temperature dependence, highlighting the presence of space charge polarization and grain boundary effects. AC conductivity analysis, modeled by Jonscher’s power law, confirmed thermally activated charge transport, with two distinct activation energies extracted from the Arrhenius plots. These two activation energies, approximately 0.18 eV and 0.37 eV, correspond to grain boundary and bulk conduction mechanisms, respectively, demonstrating the complexity of charge transport in BiBaO_3_.

The electric modulus formalism provided further insight into the dielectric relaxation processes, where the imaginary part of the modulus showed a thermally activated relaxation peak shifting toward higher frequencies with increasing temperature. This behavior confirms that the relaxation mechanisms in BiBaO_3_ are governed by localized hopping and interfacial phenomena. Magnetic measurements uncovered weak ferromagnetic behavior at room temperature, which is unusual for undoped BaBiO_3_ systems. The observed magnetism may be attributed to subtle structural distortions, oxygen non-stoichiometry, or mixed valence states of bismuth ions introduced during the sol–gel synthesis. The comprehensive analysis of BiBaO_3_ demonstrates its multifunctional nature, combining semiconducting electrical behavior with dielectric response and unexpected magnetic ordering. These results underscore the potential of sol–gel-derived BiBaO_3_ for applications in multifunctional electronic, sensing, and spintronic devices. The ability to tailor electrical and magnetic properties through synthesis parameters opens new pathways for optimizing performance in advanced material systems.

## Figures and Tables

**Table 1:** Crystallite size estimation using Scherrer equation.

Peak No.	2θ (°)	θ (°)	FWHM (°)	β (rad)	cosθ	D (nm)	hkl
1	22.3	11.15	0.0035	6.11 × 10^−5^	0.981	45.9	(100)
2	27.6	13.80	0.0028	4.89 × 10^−5^	0.972	56.4	(110)
3	31.7	15.85	0.0022	3.84 × 10^−5^	0.961	71.2	(111)
4	40.1	20.05	0.0019	3.32 × 10^−5^	0.940	80.2	(200)
5	46.5	23.25	0.0017	2.97 × 10^−5^	0.920	89.1	(210)
6	52.6	26.30	0.0015	2.62 × 10^−5^	0.898	101.2	(211)
7	57.3	28.65	0.0014	2.44 × 10^−5^	0.878	108.9	(220)
8	63.0	31.50	0.0013	2.27 × 10^−5^	0.852	117.5	(310)
9	67.1	33.55	0.0012	2.09 × 10^−5^	0.832	125.8	(311)

## Data Availability

No new data was created.
