# Correction: Tayari et al. Sol–Gel Synthesis and Comprehensive Study of Structural, Electrical, and Magnetic Properties of BiBaO_3_ Perovskite. *Gels* 2025, *11*, 450

**DOI:** 10.3390/gels12110965

**Published:** 2026-10-26

**Authors:** Faouzia Tayari, Kais Iben Nassar, João Pedro Carvalho, Sílvia Soreto Teixeira, Imen Hammami, Sílvia Rodrigues Gavinho, Manuel P. F. Graça, Manuel Almeida Valente

**Affiliations:** 1 I3N-Aveiro, Department of Physics, University of Aveiro, 3810-193 Aveiro, Portugal; faouzia.tayari@ua.pt (F.T.); jpfc@ua.pt (J.P.C.); silvia.soreto@ua.pt (S.S.T.); silviagavinho@ua.pt (S.R.G.); mpfg@ua.pt (M.P.F.G.); mav@ua.pt (M.A.V.); 2 TEMA-Centre for Mechanical Technology and Automation, Department of Mechanical Engineering, University of Aveiro, 3810-193 Aveiro, Portugal

In the original publication [1], some in-text citations were found to be insufficiently aligned with the specific statements they were intended to support. These issues were limited exclusively to citation relevance and did not affect the scientific content, data interpretation, results, or conclusions of the article.

In response to the Academic Editor’s recommendations, the authors conducted a comprehensive, fully manual, line-by-line review of the entire manuscript. Some references in Section 1 Introduction were replaced with more suitable, authoritative, and field-relevant references.

Following this correction, 25 references in the original manuscript (No. 4–21, 25, 28, 32, 54, 60, 71, and 73) were replaced by new references [2,3,4,5,6,7,8,9,10,11,12,13,14,15,16,17,18,19,20,21,22,23,24,25,26] in the same positions, while all other references remained unchanged. The total number of references is unchanged at 86. All these references have been carefully selected to ensure direct relevance to the corresponding statements in the text and have been correctly placed in the same locations as in the original publication and the current version of the paper throughout the manuscript. The same numbering and citation locations have been maintained between the published version and the current version.

All in-text citations have been manually re-mapped to ensure that each reference now directly and accurately supports the associated statement. All remaining references were thoroughly verified and confirmed to be relevant and technically justified.

No changes were made to the manuscript text, figures, tables, scientific interpretations, results, or conclusions. The scope, structure, and scientific content of the article remain identical to the originally published version. All corrections are strictly limited to citation relevance, accuracy, and traceability.

The authors state that the scientific conclusions are unaffected. This correction was approved by the Academic Editor. The original publication has been updated accordingly.
